# An Unbiased Cell‐Culture Selection Yields DNA Aptamers as Novel Senescent Cell‐Specific Reagents

**DOI:** 10.1111/acel.70245

**Published:** 2025-09-19

**Authors:** Keenan S. Pearson, Sarah K. Jachim, Caroline D. Doherty, Brandon A. Wilbanks, Luis I. Prieto, Maria Dugan, Darren J. Baker, Nathan K. LeBrasseur, L. James Maher

**Affiliations:** ^1^ Department of Biochemistry and Molecular Biology Mayo Clinic College of Medicine and Science Rochester Minnesota USA; ^2^ Department of Medicine, SMPH University of Wisconsin Madison Wisconsin USA; ^3^ The Robert and Arlene Kogod Center on Aging Mayo Clinic Rochester Minnesota USA; ^4^ Department of Orthopedic Surgery Boston Children's Hospital Boston Massachusetts USA; ^5^ Department of Molecular Pharmacology and Experimental Therapeutics Mayo Clinic College of Medicine and Science Rochester Minnesota USA; ^6^ Department of Pediatric and Adolescent Medicine Mayo Clinic Rochester Minnesota USA; ^7^ Paul F. Glenn Center for Biology of Aging Research at Mayo Clinic Mayo Clinic Rochester Minnesota USA; ^8^ Department of Physical Medicine and Rehabilitation Mayo Clinic Rochester Minnesota USA

**Keywords:** aptamer, fibronectin, in vitro selection, SELEX, senescence

## Abstract

Cellular senescence is an irreversible form of cell‐cycle arrest caused by excessive stress or damage. While various biomarkers of cellular senescence have been proposed, there are currently no universal, stand‐alone indicators of this condition. The field largely relies on the combined detection of multiple biomarkers to differentiate senescent cells from non‐senescent cells. Here we introduce a new approach: unbiased cell culture selections to identify senescent cell‐specific folded DNA aptamers from vast libraries of trillions of random 80‐mer DNAs. Senescent mouse adult fibroblasts and their non‐senescent counterparts were employed for selection. We demonstrate aptamer specificity for senescent mouse cells in culture, identify a form of fibronectin as the molecular target of two selected aptamers, show increased aptamer staining in naturally aged mouse tissues, and demonstrate decreased aptamer staining when p16 expressing cells are removed in a transgenic *INK‐ATTAC* mouse model. This work demonstrates the value of unbiased cell‐based selections to identify new senescence‐specific DNA reagents.

## Graphical abstract

Pearson et al. report the selection of DNA aptamers against senescent mouse cells, demonstrating broad binding specificity for multiple senescent mouse cell types and induction methods. Two of the aptamers bind a form of fibronectin with sub‐nanomolar affinity even in complex protein mixtures. One aptamer detects age‐ and senescence‐associated changes in mouse lung tissue, highlighting the ability of DNA aptamer selection against a senescence phenotype to generate powerful new reagents with the potential to detect or target senescent cells.

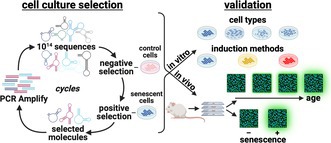

## 1 Introduction

Cellular senescence is a state of irreversible cell cycle arrest, first characterized by Hayflick and Moorhead (1961). Since that time, there has been extensive research to characterize mechanisms of senescence induction, the role of senescence in tissue remodeling, its anti‐tumorigenesis function, its contribution to age‐related diseases, and biomarkers to more specifically define senescent cells (Dodig et al. 2019; Hernandez‐Segura et al. 2018; Munoz‐Espin and Serrano 2014). While many proposed markers of senescence have been characterized, a single universal marker has not been defined. Combinations of various markers are generally needed to identify senescent cells. Common indicators include markers of cell cycle arrest, expression of cyclin‐dependent kinase inhibitors (CDKIs) p16 and p21 (Beausejour et al. 2003; Dulic et al. 1994; el‐Deiry et al. 1993; Krishnamurthy et al. 2004), expressed features of the senescence‐associated secretory phenotype (SASP) including cytokines such as IL‐6, chemokines such as MCP‐1, and metalloproteinases (Coppe et al. 2010), increased senescence‐associated β‐galactosidase (SA‐β‐gal) activity (Dimri et al. 1995), and various morphological alterations such as cell size and shape (Bayreuther et al. 1988). The accumulation of senescent cells in aging or in response to chemotherapeutic damage causes chronic inflammation due to the SASP and leads to increased pathogenesis (Campisi and d'Adda di Fagagna 2007; Krishnamurthy et al. 2004).

Senolytics are a class of drugs intended to selectively clear senescent cells. These drugs must therefore selectively target senescent cells and avoid harm to quiescent or healthy, post‐mitotic cells. To improve the targeting of senolytics, several modifications have been developed, including antibody‐drug conjugates (Poblocka et al. 2021) and lysosomal‐dependent prodrugs (Cai et al. 2020). A similar strategy has been employed using an aptamer‐functionalized liposome to target senolytic delivery and induce apoptosis specifically in fibroblast‐like synoviocytes associated with osteoarthritis, while not targeting nearby chondrocytes (Chen et al. 2022). Recently, a strategy for highly specific targeting of a subpopulation of senescent cells was reported, which employed a three‐part strategy. An aptamer targeting a membrane protein upregulated on the cells of interest was conjugated to a senolytic intended to selectively eliminate senescent cells by a self‐immolating linker cleavable by SA‐β‐gal (Xia et al. 2023).

Thus, these approaches attempt to identify markers upregulated in senescent cells of interest and modify a targeting moiety with the goal of increasing selective drug delivery. However, given the lack of universal senescent markers, there is a need for novel approaches that do not require an a priori list of candidate targets and can distinguish between senescent cells and healthy cells. With such applications in mind, here we apply Systematic Evolution of Ligands by EXponential enrichment (SELEX) to identify DNA aptamers that distinguish carefully validated senescent cells from matched, healthy cells. We characterize the binding of several candidate aptamers using various cell lines in vitro. The molecular target of two of these aptamers is identified as a form of fibronectin. Binding activity of one of the identified aptamers is shown to exhibit a correlation with age and senescence burden in vivo.

## 2 Results

### 2.1 Aptamer Selection

**FIGURE 1:**
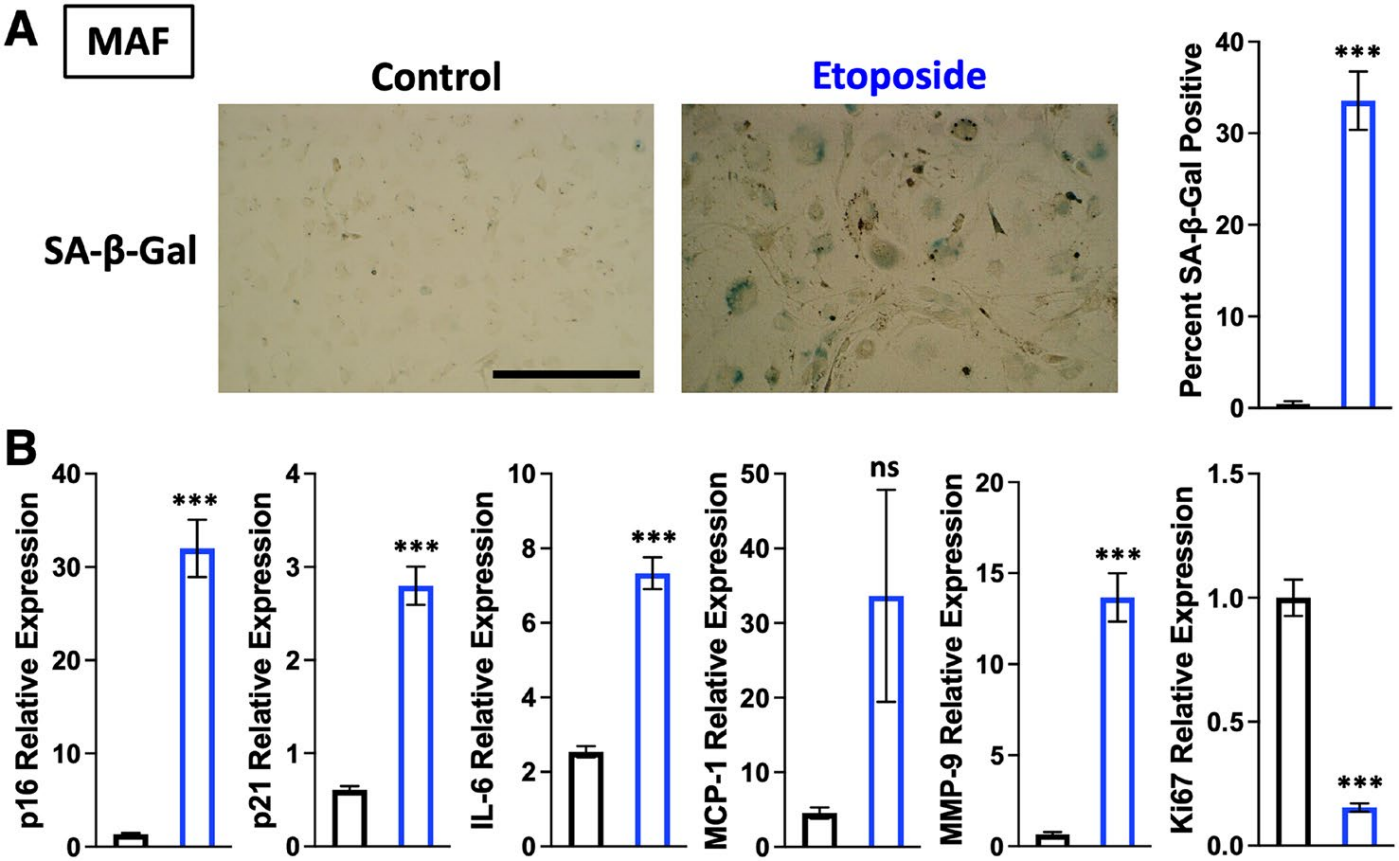
Validation of senescent phenotype in etoposide‐challenged MAFs. (A) Bright field images of control and etoposide‐challenged MAFs show morphological changes and SA‐β‐Gal staining. The percent SA‐β‐Gal positive cells is quantified per field and compared using a two‐tailed unpaired *t*‐test (*n* = 5; ***p < 0.001). Scale bar is 400 μm. (B) RT‐qPCR mRNA quantitation of various markers of senescence assessed by ΔΔCT method normalized to TATA‐box binding protein (TBP) mRNA as reference. Each comparison is made using a two‐tailed unpaired *t*‐test (*n* = 6; *p < 0.05, **p < 0.01, ***p < 0.001).

**FIGURE 2:**
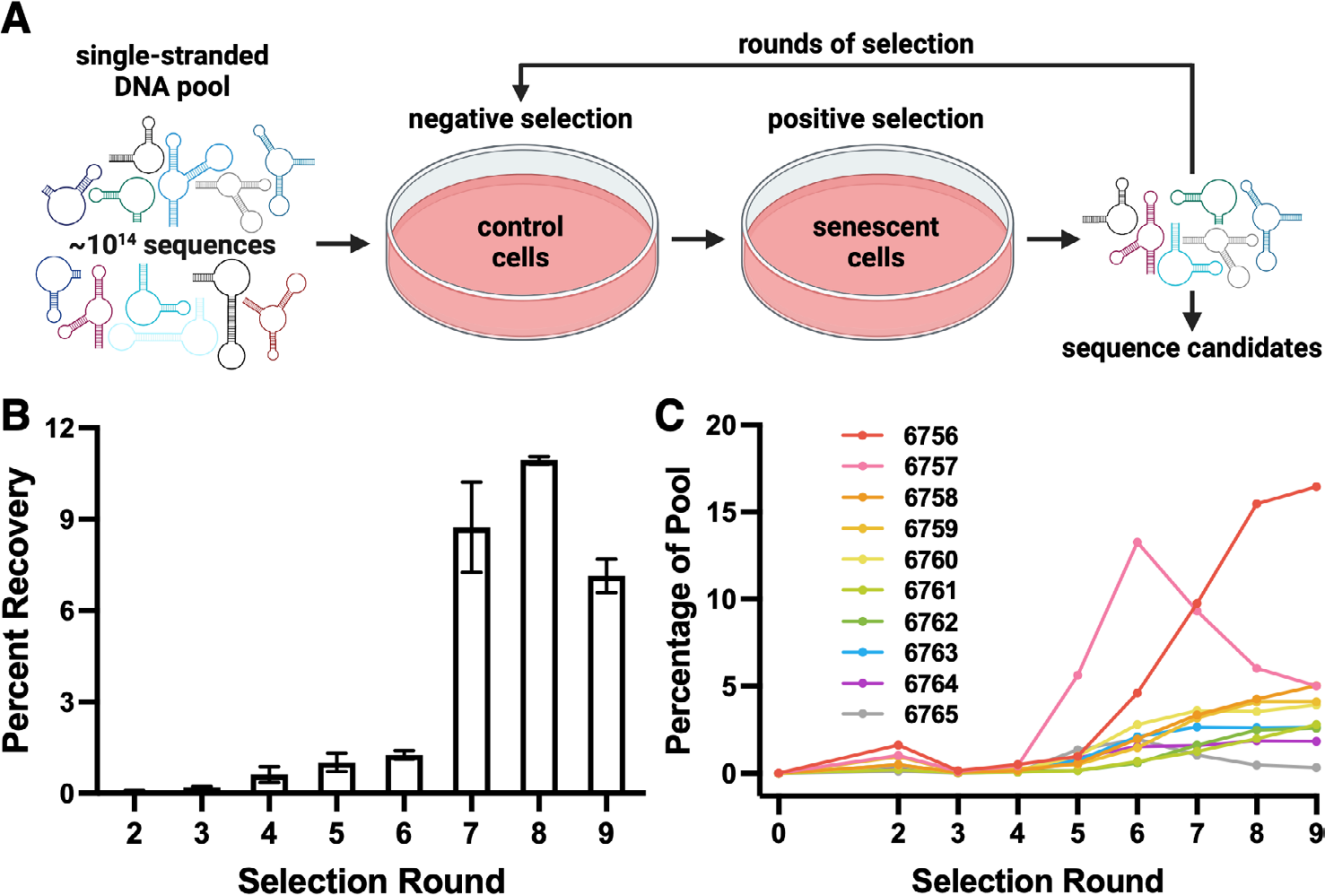
Selection of DNA aptamers that preferentially bind senescent cells. (A) Schematic of selection procedure. (B) qPCR quantification of library recovery across selection round. Error bars show standard error of technical replicates. (C) Prevalence of selected aptamer candidates in deep sequencing data across rounds of selection.

Primary mouse ear fibroblasts were isolated from adult C57BL6/J mice (henceforth termed Mouse Adult Fibroblasts, MAFs). Senescence was induced in these cells using an etoposide regimen (previously published (Jachim et al. 2023)), and the status of the MAF cultures was confirmed by change in size and morphology, increased senescence‐associated β‐galactosidase (SA‐β‐gal) activity, upregulation of cyclin‐dependent kinase inhibitors and SASP factors, and loss of the proliferation marker Ki67 (Figure 1). Unchallenged MAFs were used as negative selection “control” targets, and the etoposide‐challenged senescent MAFs served as positive selection targets (Wilbanks et al. 2025) (Figure 2A). Selection progress over multiple rounds was monitored by subjecting recovered aptamer libraries to qPCR (Pearson et al. 2022). A notable increase in library recovery was observed by round 7, and this increase was sustained through two additional rounds of selection (Figure 2B). To further assess the ability of late‐round aptamer library DNA to bind preferentially to senescent cells, the cell binding of samples of round 2 and round 8 libraries was compared. These aptamer libraries were exposed to senescent MAFs only, washed, recovered, and residual binding assessed by qPCR (Figure S1A). A significantly higher proportion of the round 8 library was recovered compared with the round 2 library (Figure S1B).

DNA aptamers from all selection rounds and the original naïve library were subjected to Next Generation Sequencing to determine candidate aptamer sequences contributing to the increased recovery observed by qPCR. The fraction of the libraries consisting of non‐unique sequences substantially increased at round 5 and continued to increase through round 9 of selection, indicating an enrichment of senescent cell specific DNA molecules (Figure S1C). We synthesized ten unrelated 80‐mer aptamer sequences and screened them for the ability to specifically bind to senescent cells (Figure 2C, Table S1). We also chose two negative control sequences present in the naïve library but not in any of the sequences recovered over 9 selection rounds (negative control oligonucleotides 6766 and 6767, Table S1).

### 2.2 In Vitro Senescent Cell Binding Screen

**FIGURE 3:**
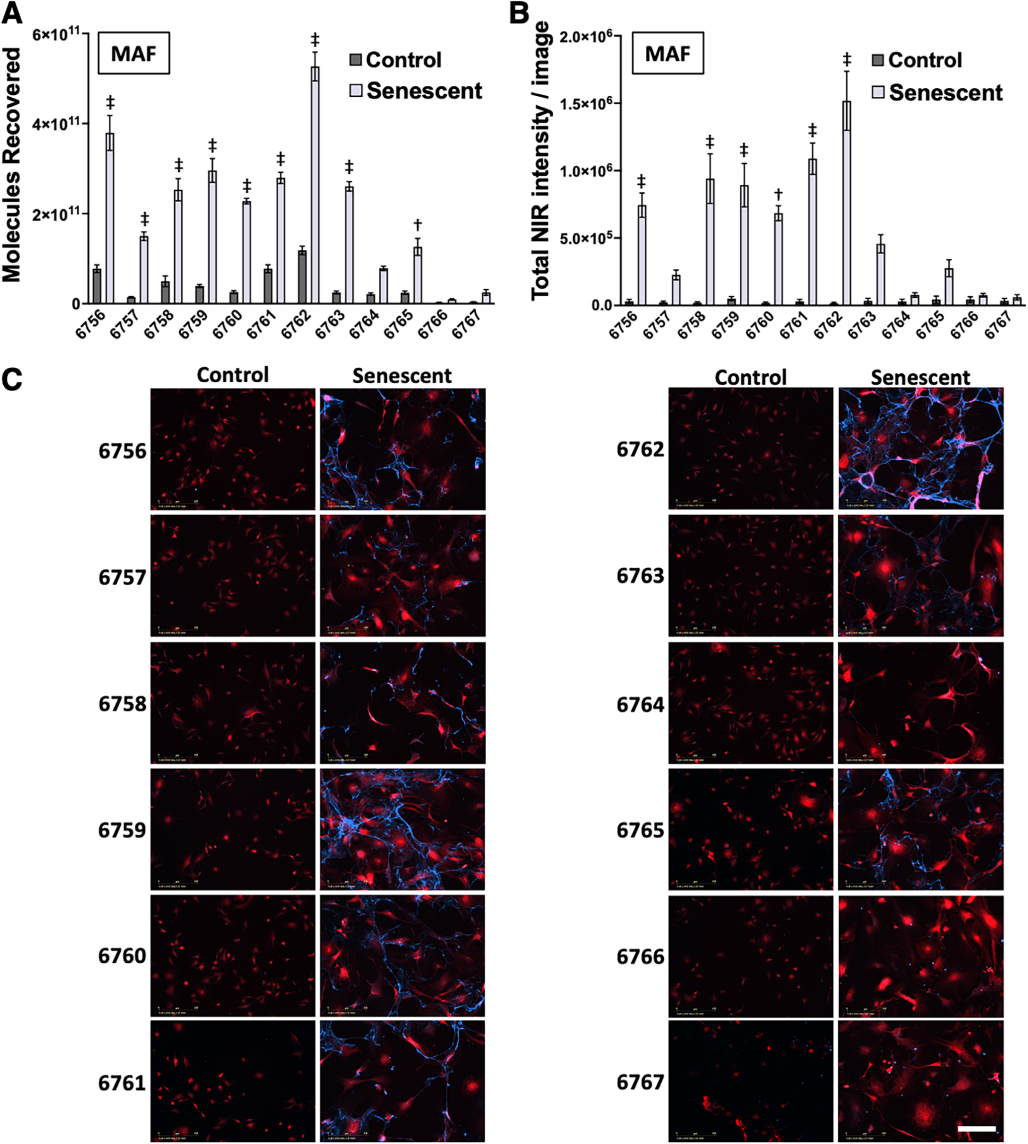
Candidate aptamer binding to senescent MAFs. (A) Quantification of candidates (6756–6765) and controls (6766 and 6767) binding to control and senescent MAFs by qPCR. Aptamer concentration was 50 nM. Statistical significance is shown for candidates compared with control 6766 by one‐way ANOVA with Dunnett's post hoc test for multiple comparisons (†: *p* < 0.005, ‡: *p* < 0.0005). Error bars indicate standard error for biological replicates (*n* = 4). (B) Quantification of staining by 50 nM aptamer/control calculated as total NIR signal intensity per image field. Statistical significance is shown for candidates compared with control 6766 by one‐way ANOVA with Dunnett's post hoc test for multiple comparisons (†: *p* < 0.005, ‡: *p* < 0.0005). Error bars are shown as standard error for multiple image fields (*n* = 9). (C) Images taken with IncuCyte SX5 are shown at 20× magnification. Constitutively expressed TdTomato is shown in red, and aptamer staining is shown in blue (secondary stain with AlexaFluor647 labeled streptavidin binding biotinylated candidates and controls). Scale bar (lower right panel) is 200 μm.

We assessed the binding of the candidate sequences and controls to senescent and control MAFs by using qPCR to monitor recovery after incubation with cells, stringent washing, and cell lysis. These results indicated that all candidate aptamers, but not negative controls, bound to senescent cells more strongly than control cells (Figure 3A, Table S2). All aptamer candidates except 6764 exhibited statistically significant senescent cell binding relative to negative control oligonucleotide 6766 (Figure 3A). We then monitored cell binding of biotin‐labeled candidates and controls using fluorescent streptavidin and quantified total fluorescence. Six of the candidates exhibited statistically significantly increased staining of senescent cells compared to 6766 (Figure 3B,C).

To assess the importance of the different culture times prior to aptamer binding for senescent cells (8 days) versus control cells (1 day), the growth of control MAFs was slowed by temporary serum starvation. An imaging experiment was repeated to assess aptamer binding under these conditions. Seven of the aptamer candidates exhibited statistically significant staining relative to negative control 6766 (Figure S2A). In contrast, when senescent MAFs were treated with trypsin and replated the day before imaging, only two aptamers exhibited statistically significant staining relative to negative control 6766 (Figure S2B). These data suggest that senescent cell‐specific aptamer binding is not an artifact due to prolonged senescent cell culture, but the data also suggest that the molecular target(s) of the selected aptamers are damaged by trypsin treatment or removed in the process of replating senescent MAFs.

Aptamer specificity was tested by subjecting MAFs to two alternative methods for senescence induction. X‐ray irradiation has been a standard for in vitro senescence induction, and we again demonstrated typical senescence markers after using this methodology (Figure S3). All tested aptamers except 6764 exhibited statistically significant binding to X‐ray‐induced senescent MAFs compared with the negative control oligonucleotide (Figure S4). Hydrogen peroxide exposure was also tested as a method for senescence induction. The resulting senescence phenotype was detectable, though less pronounced as determined by senescent marker characterization; therefore, serving as a model of oxidative stress‐induced senescence (Figure S5). Again, all the aptamers except 6764 exhibited statistically significant binding to hydrogen peroxide‐induced senescent MAFs compared with the negative control oligonucleotide (Figure S6). This demonstrates that the specificity of these aptamers for senescent cells is not changed by the induction method.

We tested the performance of candidate aptamers with a different senescent cell type. Mouse C2C12 myoblasts were challenged with etoposide, and conventional markers of senescence were assessed (Figure S7). Five of the aptamers showed significant staining of senescent C2C12 cells compared to a negative control oligonucleotide (Figure S8). These five aptamers also demonstrated specific staining of senescent C2C12 cells compared to replicating control C2C12 cells (Table S2). Similarly, mouse AML12 hepatocytes were challenged with etoposide to induce senescence (Figure S9), and we observed that five of the aptamers showed significant staining of senescent AML12 cells compared to a negative control oligonucleotide (Figure S10), as well as significant staining of senescent compared to replicating control AML12 cells (Table S2).

For further comparison, Normal Human Lung Fibroblasts (NHLF) and human IMR90 cells were challenged with etoposide, and the same increase in senescent markers was documented (Figures S11, S12). Interestingly, none of the selected aptamers bound to the senescent NHLF or IMR90 cells above the level of the negative control oligonucleotide (Figure S13). These results demonstrate that the selected aptamers display binding specificity for different senescent mouse cells (MAFs, C2C12, and AML12) compared to senescent human cells (NHLF, IMR90).

## 3 Binding Target Identification and Characterization

To identify possible molecular targets, aptamers 6756 and 6762 were chosen for analysis. A SILAC‐based proteomic assay was performed as described in Materials and Methods. Briefly, potential target proteins on isotopically labeled cultured cells were cross‐linked to the tested biotinylated aptamer (6756 or 6762) using formaldehyde. A biotinylated negative control oligonucleotide, 6766, was similarly cross‐linked but to non‐isotopically labeled cells. Cell lysates were applied to streptavidin magnetic beads, followed by stringent washing. Proteins were recovered by cross‐link reversal at elevated temperatures. Heavy and light isotope labeled samples were combined as aptamer and negative control. Experiments were repeated with heavy and light isotope labeling reversed. After trypsin digestion, samples were subjected to LC–MS/MS analysis and peptide mass fingerprinting to identify proteins uniquely cross‐linked to specific aptamers. Notably, peptides from only a single protein, fibronectin, were strongly cross‐linked by either aptamer 6756 or 6762, displaying a selection ratio > 1 for duplicate forward (heavy + aptamer/light + control) and duplicate reverse (light + aptamer/heavy + control) experiments (Table 1).

**TABLE 1:** Results from SILAC‐based proteomics experiment show only fibronectin enriched in either 6756 or 6762 compared with negative control 6766.

Protein ID	Protein name	Gene name	Unique peptides	Ratio (6756/6766)	Ratio (6762/6766)
P11276	Fibronectin	FN1	35–56	1.80 ± 0.41	1.85 ± 0.20

**FIGURE 4:**
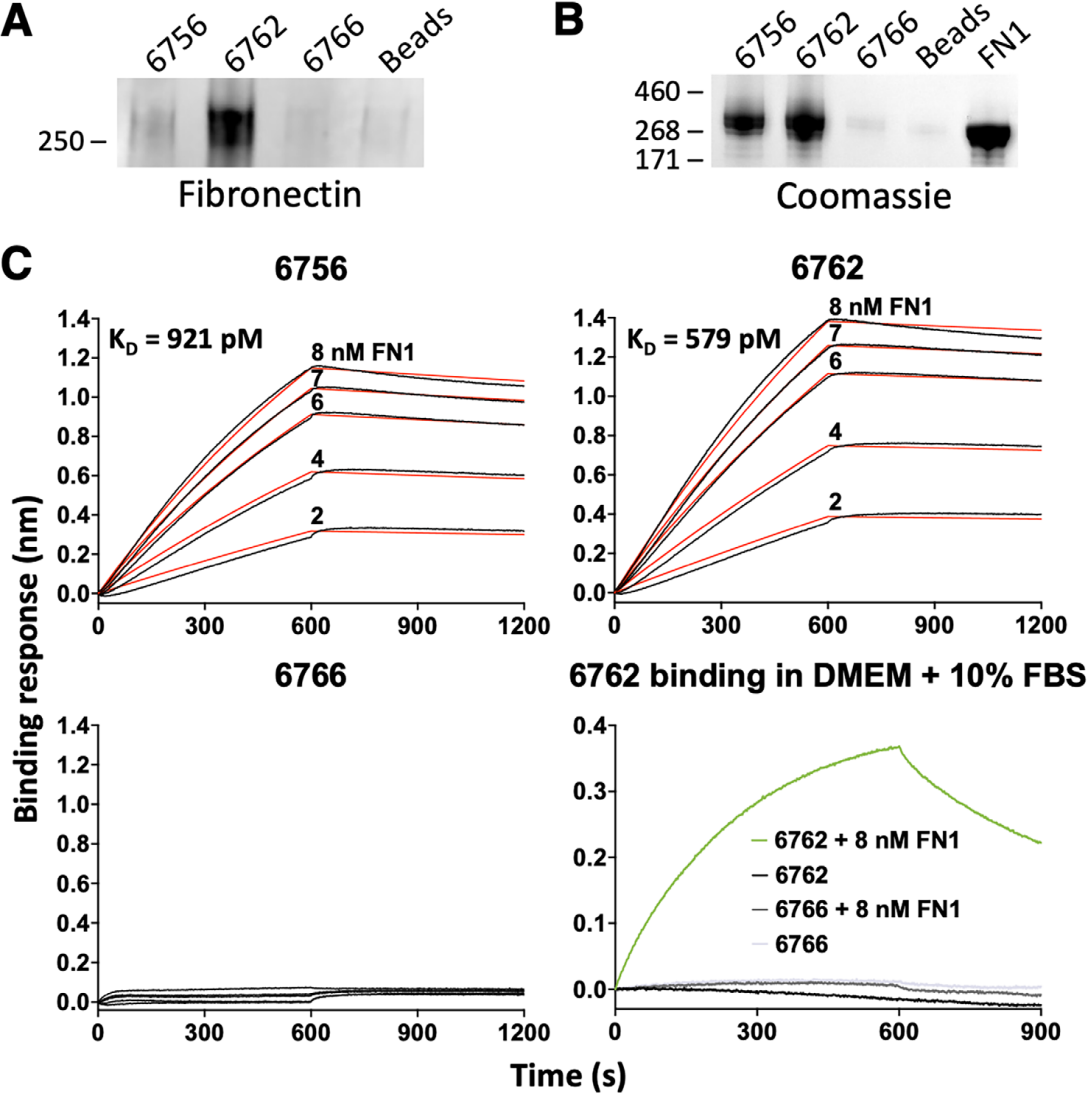
Identification and verification of target protein of aptamers 6756 and 6762. (A) Aptamer pull‐down from cell lysates followed by SDS‐PAGE and Western blotting shows fibronectin enrichment with aptamers 6756 and 6762 compared to negative control 6766 and bead‐only control. (B) Purified fibronectin from mouse plasma was incubated with biotinylated aptamer or negative control loaded streptavidin magnetic beads. After washing, bound protein was eluted, separated by SDS‐PAGE, and visualized by Coomassie blue staining. (C) Biolayer Interferometry shows high‐affinity binding of aptamers 6756 (*K*_D_ = 921 pM) and 6762 (*K*_D_ = 579 pM) to purified fibronectin, with no binding detected for negative control 6766. Sensograms (black lines) and fitted binding models (red lines) are shown for each aptamer‐protein interaction. Additional analysis demonstrates that 6762 binds fibronectin in the presence of DMEM containing 10% FBS.

To validate this nominated molecular binding partner, we performed the same cross‐link and pull‐down approach described above, followed by a Western blot. This experiment showed that aptamers 6756 and 6762 selectively enriched fibronectin compared to the negative control oligonucleotide 6766 or streptavidin magnetic beads only (Figures 4A and S14). Further, purified native mouse fibronectin was exposed to immobilized aptamer or negative control oligonucleotide displayed on streptavidin magnetic beads without formaldehyde cross‐linking. This procedure resulted in fibronectin capture by aptamers 6756 and 6762 when compared with the negative control oligonucleotide 6766 or beads alone (Figure 4B).

The affinity of 6756 and 6762 for fibronectin was assessed by biolayer interferometry (BLI). Aptamers 6756 and 6762 exhibited equilibrium dissociation constants of 921 pM and 579 pM, respectively (Figure 4C). The negative control oligonucleotide 6766 displayed no appreciable binding (Figure 4C). Aptamer 6762 binding to fibronectin was further tested in a more complex environment consisting of DMEM + 10% FBS, and the specificity of binding was demonstrated against the negative control 6766 (Figure 4C).

Confocal microscopy was used to assess colocalization of anti‐fibronectin antibody staining and staining by aptamers 6756 or 6762 on cultured cells. Interestingly, the staining patterns are subtly different, suggesting that a variant form of fibronectin may be detected by senescent cell‐specific aptamers (Figure S15). Confocal microscopy was also utilized to assess the relative amount of anti‐fibronectin antibody staining on control and senescent mouse C2C12 cells and human IMR90 cells. Both cell lines showed an increased amount of fibronectin staining in senescent cells versus control cells. There was not a statistically significant difference in the amount of fibronectin staining between senescent C2C12 and senescent IMR90 cells (Figure S16A,B). Additionally, we confirmed that fibronectin protein increases in total protein lysates from senescent MAFs compared with control cultures by Western blot (Figure S16C,D).

Given that cellular staining with aptamers 6756 and 6762 does not precisely match that of conventional anti‐fibronectin antibodies, one possibility is that 6756 and/or 6762 have specificity for fibronectin containing Extra Domain A (EDA). The peptides detected in the LC–MS/MS experiment were mapped to the full fibronectin amino acid sequence, showing that peptides unique to Extra Domain A were detected (Figure S17).

Levels of *Fn1* mRNA transcripts (encoding fibronectin) from RNA‐seq data were compared between etoposide‐induced senescent MAFs and control MAFs. Total *Fn1* transcript expression is not statistically different between the two in vitro conditions, while quantification of only those transcripts encoding for EDA‐containing fibronectin is statistically different (Figure S18).

## 4 Aptamer Stain Correlation With In Vivo Age and Senescence Burden

**FIGURE 5:**
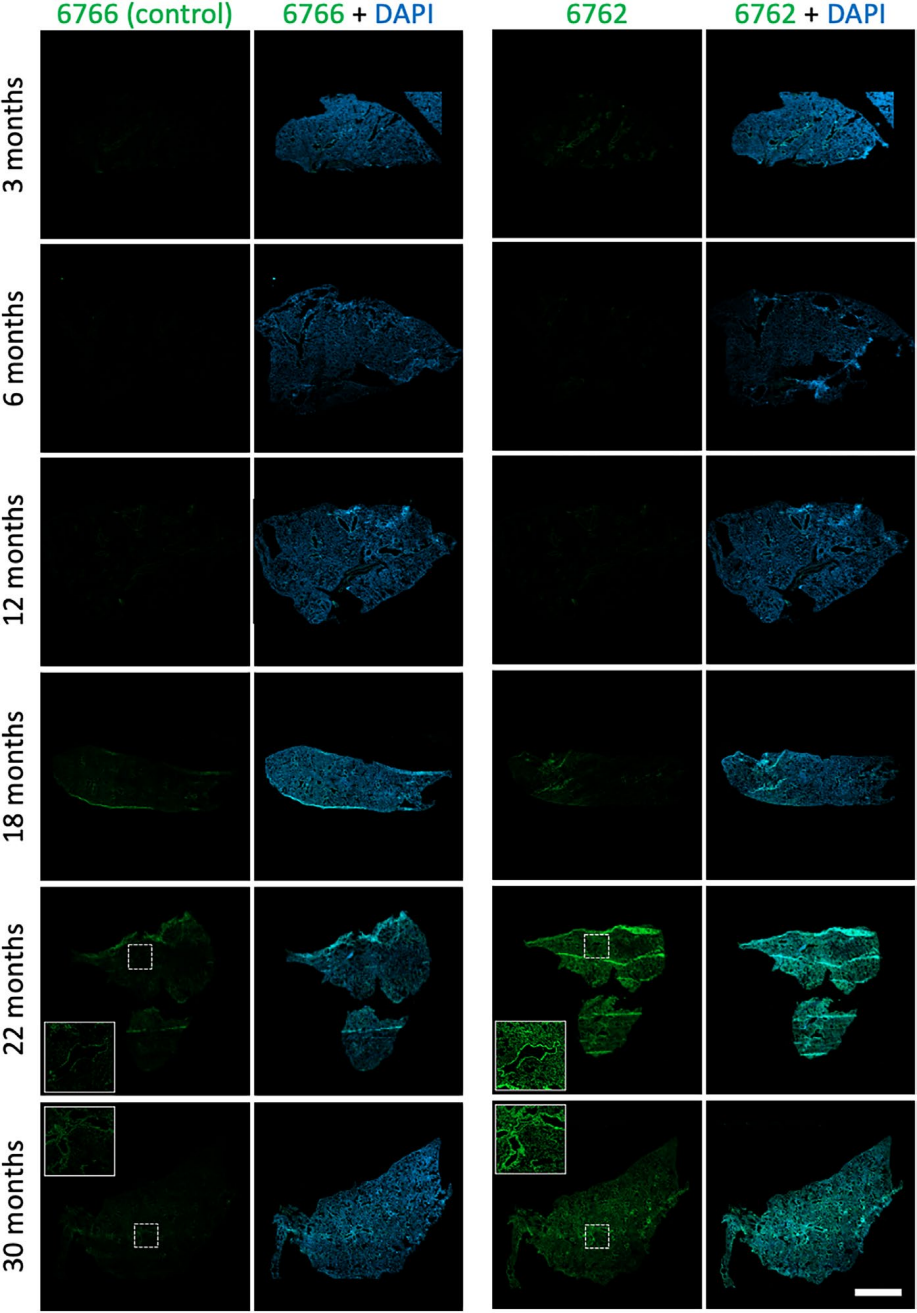
Aptamer 6762 staining of mouse lung tissue increases with age. Aptamer 6762 and negative control oligonucleotide 6766 are directly detected using the fluorescein label (green). Nuclei are stained with DAPI (blue). Inlays are 3× enlargements of indicated regions. The images are 5× tiled confocal images of the entire tissue section. Scale bar (lower right panel) is 2 mm.

To assess whether DNA aptamer specificity for senescent cells in vitro correlates with binding to tissue from animals of different ages, we compared senescence‐specific aptamer 6762 to negative control 6766 for staining lung tissue sections from naturally aged mice. There is no appreciable staining in younger mice, and we observed that for ages 22 and 30 months there is an obvious increase in specific aptamer staining across the tissue sections that is much stronger than for the negative control (Figure 5). Higher magnification images show that this staining varies by region of the tissue (Figure S19). This increased staining with age is consistent with the known age‐associated increase in senescent cell burden.

**FIGURE 6:**
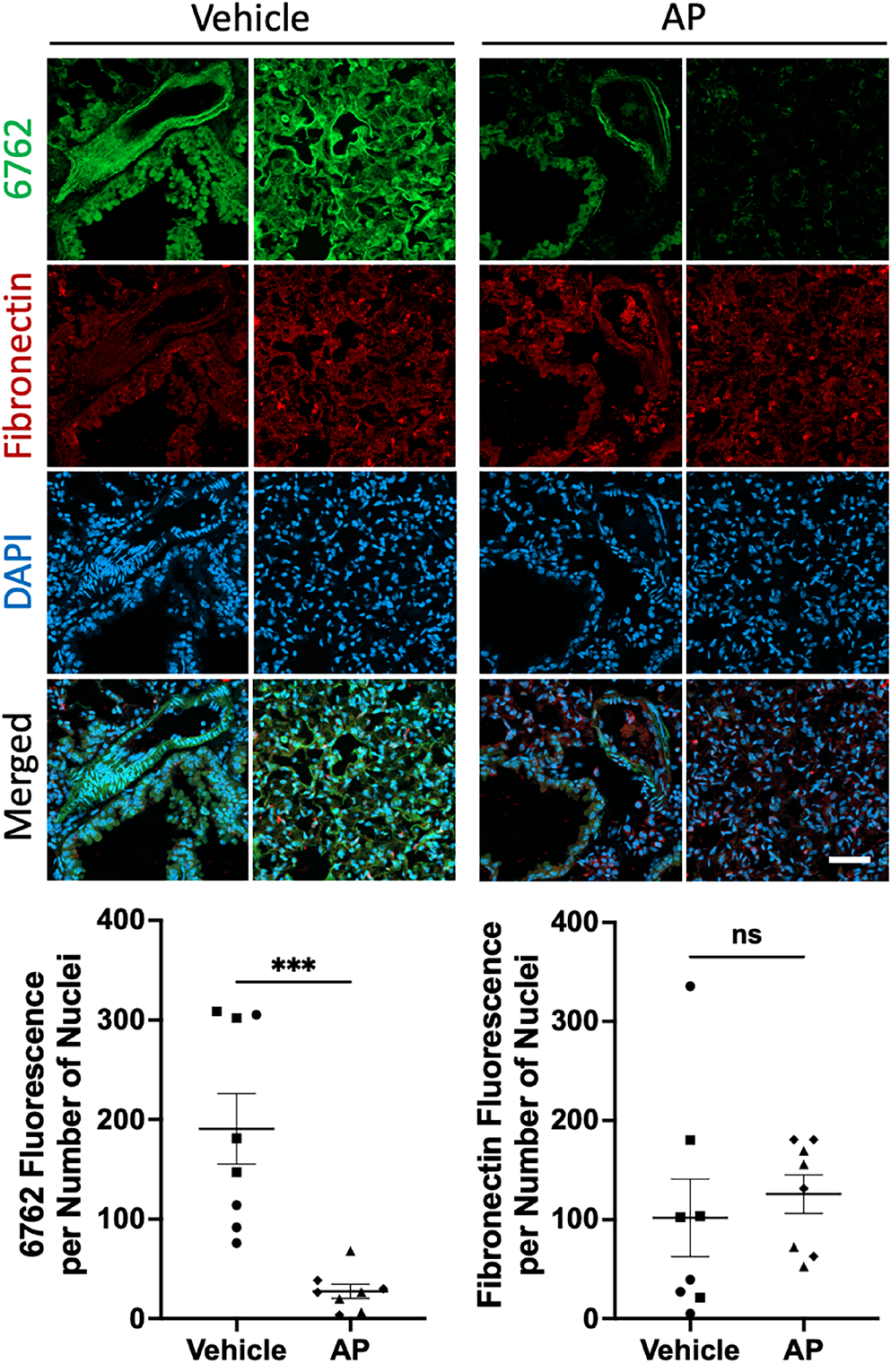
Removal of p16‐positive cells leads to reduced aptamer 6762 (but not fibronectin) staining. Aptamer 6762 is detected by fluorescein label (green). Fibronectin antibody is detected with AlexaFluor594 anti‐rabbit secondary (red). Nuclei are stained with DAPI (blue). Quantification of aptamer 6762 fluorescence per nuclei and fibronectin fluorescence per nuclei was obtained from 8 image fields per condition. Each comparison is made using a two‐tailed unpaired *t*‐test (***p < 0.001, ns: *p* > 0.05). Scale bar (lower right panel) is 50 μm.

To further assess whether age‐related staining of tissue sections correlates with in vivo senescent cell burden, we stained lung tissues from 21‐month‐old *INK‐ATTAC* mice either treated with AP (selectively eliminates senescent cells (Baker et al. 2016)) or with vehicle. AP‐treated tissues showed a statistically significant decrease in 6762 staining compared with vehicle (Figures 6, S20, and S21). In this same experiment, there was no change in anti‐fibronectin antibody staining (Figures 6, S20, and S21). Interestingly, higher magnification (40×) images show that, as in the in vitro experiment, anti‐fibronectin antibody and 6762 stains do not show the same pattern of target interaction (Figure S22).

Picrosirius red (PSR) staining of lung sections was used to evaluate extracellular matrix changes following clearance of p16‐positive cells in *INK‐ATTAC* mice. Compared with vehicle‐treated animals, AP treatment resulted in visibly reduced collagen deposition (Figure S23). To further explore the relationship between aptamer binding and senescence in vivo, we examined aptamer 6762 staining in lung tissue from 24‐month‐old p16Cre; Ai14 mice (Cherry et al. 2023). Aptamer 6762 staining was more extensive than the tdTomato signal marking p16‐positive cells, suggesting that aptamer recognition encompasses a broader population than those defined solely by p16 expression (Figure S24).

## 5 Discussion

The results reported here suggest the successful identification of a collection of DNA aptamers with varying specificities for senescent cells in culture. These results extend to various cell types (fibroblasts, myoblasts, and hepatocytes) and various methods for senescence induction (etoposide challenge, X‐ray irradiation, and hydrogen peroxide exposure). Our results further suggest that the selected aptamers demonstrate a binding preference for senescent mouse cells over senescent human cells. This selectivity might be explored by screening additional cell lines or epitope mapping of target binding. The apparent fibronectin specificity of lead‐selected aptamers is interesting considering the mouse vs. human selectivity and the evident difference in cell staining patterns by selected aptamers and conventional anti‐fibronectin antibodies. It may be possible to review epitope differences and post‐translational modifications that vary between mouse and human fibronectin to understand these results. Because the staining patterns between the anti‐fibronectin antibody and aptamers 6756 (in vitro) and 6762 (in vitro and in vivo) do not match, one potentially contributing factor is that the smaller size of DNA aptamers results in greater tissue section penetration. Another likely possibility is that the aptamer target epitope may represent a unique fibronectin form produced during senescence.

Recent studies have found conflicting results regarding the role of fibronectin in aging and senescence. Fibronectin type III domain‐containing protein 5 (FNDC5) is a strong candidate biomarker as it is protective against many age‐associated pathologies (Cardoso et al. 2018). Further, culturing adipocyte‐derived stem cells on a fibronectin coating reduces stress‐associated pathways and alleviates senescence (Tragoonlugkana et al. 2024). On the other hand, fibronectin exacerbated senescence in dermal fibroblasts (Perie et al. 2024), and different isoforms were identified with increasing passage. FN‐EDA1 (fibronectin containing extra domain A) is a unique splice variant more highly expressed in tissues with higher senescence burden (Sueblinvong et al. 2014). In the ECM, it was found to be particularly detrimental to the pathophysiology of atherosclerotic plaques (Doddapattar et al. 2020). Our data suggest that our selected aptamers are binding to senescence‐associated FN‐EDA1, as we see peptides unique to this domain present in our LC–MS/MS data and a statistical difference in transcripts encoding this isoform between control and senescent fibroblasts. This concept is further supported by our results showing that fibronectin expression is constant in senescent mouse and senescent human cells in culture, as judged by an anti‐fibronectin antibody, indicating that what is recognized by this conventional reagent is not the identical epitope recognized by these aptamers.

In our study, we noted an increase in fibronectin protein abundance in senescence, consistent with previous reports (Kumazaki et al. 1991), along with increased staining with an anti‐fibronectin antibody. However, this is not accompanied by an increase in total fibronectin mRNA, which was seen in other previously published work (Casella et al. 2019). Interestingly, we did not see a correlation with anti‐fibronectin antibody staining and senescence burden. We interpret our results to further show that some unique senescence‐specific form of fibronectin, potentially FN1‐EDA, is detected during the selection of multiple aptamers in our unbiased cell culture selection. However, this would not explain the difference in species specificity between the reagents. Given the variable performance of our selected aptamers in different biological contexts (different in vitro cell types and in vivo tissues), this suggests that alternative splicing of fibronectin into different isoforms is spatially and temporally variable, a complexity which our homogenous in vitro selection method did not fully recapitulate. While our selection approach identified potentially useful targets, with potential future applications as biomarkers, potential diagnostic/prognostic markers, and possible targets for homing senolytics, further testing will be needed to judge their viability as universal senescence markers.

Aptamer 6762 stains widely across all mouse tissue sections associated with advanced age and senescent cell burden. If 6762 is truly selective for senescent cells, this staining result would indicate a much higher prevalence of senescent cells than previous work has shown in animals at advanced age (Biran et al. 2017). Because 6762 binds some form of fibronectin, a component of the extracellular matrix (ECM), we hypothesize that some of the observed staining may not directly indicate the presence of senescent cells, but perhaps ECM residue left behind by senescent cells that have migrated or been cleared over time. Additionally, the powerful paracrine signaling from the SASP of senescent cells can remodel ECM and change the local tissue environment, even when senescent cells are rare. This concept is supported by our work, which stained lung tissue from mice expressing a p16 reporter. This analysis showed a relatively low number of p16‐positive cells in comparison to the extensive staining by aptamer 6762. Further, through PSR staining we showed that collagen deposition in the ECM of aged lung tissues exhibits variability. However, it appears to decrease when senescent cell accumulation is limited, highlighting the importance of considering ECM and tissue microenvironments in identifying age‐related or senescent‐related targets in vitro.

It is possible to envision future aptamer selections to identify aptamers for senescent human cells. The robustness of the selection process could be enhanced by including different cell lines, alternated through the rounds of selection, to improve the universal potential of identified aptamers across multiple tissues. Additionally, replicative senescent cells could be used with, or instead of, stress‐induced senescent cells, or different senescence‐induction methods could be alternated through rounds of selection (Toussaint et al. 2000). Further, since ECM components are highly abundant and readily accessible to aptamers, controlling for seeding density and time in culture will be crucial factors for future SELEX experiments. These considerations will further improve the specificity to different senescent cells and improve in vivo specificity. Selections might also be designed to identify aptamers that are internalized into senescent cells. Such tools might eventually be applied prognostically to identify individuals most likely to benefit from senolytic treatment and might be applied in the development of aptamer‐drug conjugates that selectively deliver drugs to senescent cells. Such approaches might improve the specificity of senolytics and senomorphics.

The complexity of the senescence phenotype and the lack of a single universal biomarker to reliably identify senescent cells have posed significant challenges in the field of aging research. In this study, we leveraged an unbiased cell‐based selection technique and demonstrated its value in identifying new senescence‐specific DNA reagents. Through further refinement of the selection process, SELEX could potentially be used to identify senescence markers more universal than those currently available in the field of aging biology.

## Author Contributions

Conceptualization: K.S.P., S.K.J., N.K.L., L.J.M. Funding acquisition: D.J.B., N.K.L., L.J.M. Investigation: K.S.P., S.K.J., C.D.D., B.A.W., L.I.P., M.D. Data analysis: K.S.P., S.K.J., C.D.D., B.A.W., L.I.P., M.D. Visualization: K.S.P. Writing – original draft: K.S.P., L.J.M. Writing – review and editing: K.S.P., S.K.J., C.D.D., B.A.W., L.I.P., D.J.B., N.K.L., L.J.M. All authors read and approved the final manuscript.

## Conflicts of Interest

D.J.B. has a potential financial interest related to this research. He is a co‐inventor on patents held by Mayo Clinic, patent applications licensed to or filed by Unity Biotechnology, and a Unity Biotechnology shareholder. Research in the Baker laboratory has been reviewed by the Mayo Clinic Conflicts of Interest Review Board and is being conducted in compliance with Mayo Clinic Conflicts of Interest policies. The other authors declare no conflicts of interest.

## Supporting information


**Appendix S1:** acel70245‐sup‐0001‐AppendixS1.pdf.

## Data Availability

All data supporting the statements and conclusions made by the authors are included in the figures and in the Supporting Information.
